# Using self-determination theory to promote adolescent girls' physical activity: Exploring the theoretical fidelity of the Bristol Girls Dance Project

**DOI:** 10.1016/j.psychsport.2016.01.009

**Published:** 2016-05

**Authors:** Simon J. Sebire, Joanna M. Kesten, Mark J. Edwards, Thomas May, Kathryn Banfield, Keeley Tomkinson, Peter S. Blair, Emma L. Bird, Jane E. Powell, Russell Jago

**Affiliations:** a Centre for Exercise, Nutrition & Health Sciences, School for Policy Studies, University of Bristol, 8 Priory Road, Bristol, BS8 1TZ, UK; b Bristol Randomised Trials Collaboration, School of Social & Community Medicine, University of Bristol, Bristol, BS8 2PS, UK; c Health and Social Sciences, University of the West of England, Bristol, UK

**Keywords:** Self-determination theory, Process evaluation, Intervention, Physical activity, Adolescents

## Abstract

**Objectives:**

To report the theory-based process evaluation of the Bristol Girls' Dance Project, a cluster-randomised controlled trial to increase adolescent girls' physical activity.

**Design:**

A mixed-method process evaluation of the intervention's self-determination theory components comprising lesson observations, post-intervention interviews and focus groups.

**Method:**

Four intervention dance lessons per dance instructor were observed, audio recorded and rated to estimate the use of need-supportive teaching strategies. Intervention participants (n = 281) reported their dance instructors' provision of autonomy-support. Semi-structured interviews with the dance instructors (n = 10) explored fidelity to the theory and focus groups were conducted with participants (n = 59) in each school to explore their receipt of the intervention and views on the dance instructors' motivating style.

**Results:**

Although instructors accepted the theory-based approach, intervention fidelity was variable. Relatedness support was the most commonly observed need-supportive teaching behaviour, provision of structure was moderate and autonomy-support was comparatively low. The qualitative findings identified how instructors supported competence and developed trusting relationships with participants. Fidelity was challenged where autonomy provision was limited to option choices rather than input into the pace or direction of lessons and where controlling teaching styles were adopted, often to manage disruptive behaviour.

**Conclusion:**

The successes and challenges to achieving theoretical fidelity in the Bristol Girls' Dance Project may help explain the intervention effects and can more broadly inform the design of theory-based complex interventions aimed at increasing young people's physical activity in after-school settings.

## Highlights

• A comprehensive qualitative and quantitative process evaluation is reported.
• In particular, we focus on evaluating the theory that the intervention was based on.
• The results can inform future self-determination theory interventions.

## 1 Introduction

Young people become less active during the transition from childhood to adolescence (Nader, Bradley, Houts, McRitchie, & O'Brien, 2008). Girls are less active and experience a steeper decline in activity than boys (Nader et al., 2008). In England, the majority of adolescent girls do not meet the government's recommendations of a minimum of 60 min of moderate-to-vigorous physical activity (MVPA) per day (Joint Health Surveys Unit, 2013). As physical activity is associated with physical and mental health (Janssen & Leblanc, 2010), identifying ways to encourage more girls to be active more often is a national (Department of Health, 2011) and global (World Health Organisation, 2004) health promotion priority. A recent meta-analysis has shown that physical activity interventions for girls are more effective if they exclude boys, are delivered at school and are based on an underlying theory of behaviour change (Pearson, Braithwaite, & Biddle, 2015).

Dance is a popular activity amongst girls (O'Donovan & Kay, 2005) and proliferates contemporary culture and media consumed by young people such as music TV, talent shows and singing contests. Dance can be an enjoyable form of cardiovascular exercise in which girls develop their co-ordination, acquire new skills, work independently and in groups and develop friendships and self-expression (Australian Women Sport and Recreation Association, 2010). Dance is an alternative to traditional/competitive sports offered to girls and we have previously highlighted the potential of a dance-based physical activity intervention for adolescent girls: the Bristol Girls Dance Project (BGDP) (Jago et al., 2013, Jago et al., 2012, Jago et al., 2011, Powell et al., 2013). The BGDP was a cluster-randomised controlled trial designed to examine the effectiveness and cost-effectiveness of an after-school dance-based intervention in increasing the MVPA of Year 7 girls (aged 11–12 years).

### 1.1 Theoretical foundations of BGDP

Underpinning interventions with behavioural theory is hypothesised to increase their effectiveness (Baranowski et al., 1998, Craig et al., 2008). In addition, theories allow intervention developers to target activities at theoretically-derived mediators (Baranowski et al., 1998). The BGDP intervention was based on self-determination theory (SDT) (Deci and Ryan, 2000, Ryan and Deci, 2007) because its theoretical foundations are concerned with how the psychological and socio-environmental conditions (e.g., created by a dance teacher) can support individuals' motivation (Fortier, Duda, Guerin, & Teixeira, 2012).

### 1.2 Motivation quality

According to SDT, an individual's motivation for a behaviour such as dance, can be more or less self-determined and six different types of motivation are hypothesised to be differently associated with behaviours such as physical activity and related cognitive and affective outcomes (Ryan & Deci, 2007). The more self-determined types of motivation (i.e., intrinsic motivation, integrated & identified behavioural regulation) are broadly grouped as *autonomous*. Intrinsic motivation is based on the inherent satisfaction or enjoyment that accompanies a given behaviour. The other forms of autonomous motivation are extrinsic in nature and involve undertaking a behaviour for a reason other than its inherent satisfaction. Integrated regulation is where a person aligns their engagement in a behaviour with their broader self (e.g., seeing being active as part of one's identity) and identified regulation represents motivation which is driven by a valued outcome such as health benefits or making new friends. The less self-determined types of motivation (i.e., introjected & external regulation) are broadly grouped as *controlled* motivations. Introjected regulation refers to motivation based on internalised pressures such as avoiding feelings of guilt, whereas external regulation is characterised by prods and pushes which are external to the person such as complying with demands or avoiding punishments. Previous research suggests that more autonomous physical activity motivation is positively associated with child and adolescent physical activity (Owen et al., 2014, Sebire et al., 2013) and positive psychological outcomes such as quality of life and physical self-concept (Standage, Gillison, Ntoumanis, & Treasure, 2012). On the other hand, adolescents' controlled motivation for exercise has been shown to correlate negatively with health-related quality of life and functioning within physical, social, school and emotional domains (Standage et al., 2012).

### 1.3 Fostering high quality motivation

A cornerstone of SDT is that autonomous motivation is developed when people feel that their psychological needs for autonomy (i.e., feelings of volition and free will), competence (i.e., feeling capable to perform challenging tasks) and relatedness (i.e., perceptions of belonging & meaningful connections with others) are fulfilled (Deci & Ryan, 2000). This hypothesis is supported by empirical research among children (Sebire et al., 2013), adolescents (Van den Berghe, Vansteenkiste, Cardon, Kirk, & Haerens, 2014) and adult dancers (Quested & Duda, 2010). Within SDT, people's psychological needs can be supported or undermined by the motivational climate that an authority figure (e.g., dance instructor) creates through their motivating or teaching style (Deci and Ryan, 2008, Su and Reeve, 2011). Need supportive styles are underpinned by the provision of autonomy support, structure and involvement which is reflected in how teachers' (or dance instructors) conduct their classes and interact with pupils (Haerens et al., 2013, Su and Reeve, 2011). When teachers provide autonomy support they give meaningful rationales (especially for tasks which are important but not as enjoyable as others), offer choices which pupils value, seek and acknowledge pupils' perspectives or ideas and nurture pupils' internal motivation, interest and enjoyment. In contrast, controlling teachers aim to motivate pupils by either inducing internal pressures such as guilt, or external pressure such as a deadline and feedback given and language is used to manipulate rather than be informative. Such strategies are likely to frustrate rather than support pupils' psychological needs (Bartholomew, Ntoumanis, & Thogersen-Ntoumani, 2009). Teacher's provision of structure is primarily related to supporting pupil's competence. A well-structured class is where clear expectations are set out before tasks and during tasks, guidance, direction and positive effect-based feedback is given. Without structure, a learning environment can be described as chaotic where students do not know what they should do or what is expected of them (Vansteenkiste et al., 2012). Pupils' relatedness is supported when teachers' are involved by showing the pupils empathy and genuine interest in them (Aelterman et al., 2013, Haerens et al., 2013, Su and Reeve, 2011). In contrast, a lack of involvement by teachers will frustrate relatedness. Amongst children, Physical Education (PE) teachers' use of need-supportive styles has been shown to be associated with their pupils' psychological need satisfaction and autonomous motivation for PE (Ntoumanis and Standage, 2009, Van den Berghe et al., 2014).

### 1.4 Design of the BGDP

We have previously reported the study protocol (Jago et al., 2013) and outcome paper (Jago et al., 2015). The study involved 571 Year 7 girls (aged 11–12 years) from 18 schools from the greater Bristol area allocated at the school-level to intervention (n = 9) and control (n = 9) arms. The intervention consisted of 40, 75-min after-school lessons that took place, twice per week for 20 weeks at school and were led by 10 professional dance instructors between January and July 2014. Girls were provided with a dance diary which they could complete and hand in to the dance instructor at the end of each lesson, in which they could record what they had learnt, their feelings and thoughts. Instructors were provided with a manual which provided plans for all 40 lessons in addition to training outlined below. One instructor was unable to complete the full intervention and was replaced at the intervention mid-point with another instructor. One instructor taught in two schools (ID numbers 21 and 51).

### 1.5 Embedding SDT within the BGDP intervention

BGDP aimed to increase girls' autonomous motivation for both dance and physical activity and this was targeted through the BGDP instructor manual, the lesson design and content, and dance instructor intervention training (Table 1). Dance instructors received a one day training session (5th December 2013) prior to the start of the intervention (13th January 2014) which included 2 h on SDT (delivered by SJS) highlighting the key features of the training manual and how the theory could be applied in dance lessons. The content of the manual and the training focussed on how to provide autonomy, competence and relatedness support and intertwined involvement and structure as ways to achieve this. Comparisons were made between using these need-supportive strategies and more controlling practices. Instructors were given the opportunity to practice using need-supportive techniques by role-playing different dance activities, asking questions and receiving feedback. At the mid-point of the intervention, instructors attended a half-day top-up training session where the SDT components were revisited and instructors shared their experiences of delivery to resolve any problems.

### 1.6 Results of BGDP and the need for a theoretical process evaluation

The BGDP intervention was not effective in increasing girls' MVPA at the end of the intervention period when dance lessons were still running or at 12 months after baseline (Jago et al., 2015). Following the intervention, autonomous and controlled motivation and perceptions of competence and relatedness were lower among intervention versus control group participants. This was unexpected and highlighted the importance of using process evaluation to further understand these findings and the motivational processes at play. The part of the BGDP process evaluation which focussed on the dose of the intervention received (i.e. the number of lessons attended) and evaluation of issues pertaining to attendance and retention identified that between 37 and 40 dance lessons were delivered in all schools (Sebire et al.,). Attendance (*M* = 12.8, SD = 7.0) at dance lessons declined over time and approximately nine girls per school out of a possible 33 received the intervention dose (defined as two thirds of the lessons offered). Participants enjoyed taking part and reported social and physical health benefits. Instructors valued the intervention training and identified that variable attendance challenged delivery but also facilitated the development of a core group of committed participants. Multiple process evaluation papers are justified to make full use of all of the data collected (Moore et al., 2014). Given the large volume of data produced from the detailed process evaluation of BGDP the aim of this paper is to report a process evaluation of the BGDP study with particular focus on theoretical fidelity. Process evaluation can play a crucial role in illuminating how theory-based intervention components are experienced by the participants and importantly how they are received, interpreted and implemented in practice by intervention deliverers. The findings can then be used to interpret findings of intervention effects in greater detail and refine how theory can be best operationalised in practice.

Specifically we sought to use both quantitative and qualitative methods to evaluate: (a) the degree to which dance instructors adopted a need-supportive style, (b) participants' perceptions of the dance instructors' teaching practices, (c) participants' qualitative perceptions of satisfaction of autonomy, competence and relatedness and (d) dance instructors' experiences of delivering an SDT-based intervention.

## 2 Methods

### 2.1 Data collection

#### 2.1.1 Qualitative data collection

At the end of the intervention and within three weeks of the final dance lesson (delivered 11th July 2014), the 10 dance instructors participated in individual semi-structured interviews with JMK (mean duration = 67.2 min, range = 41.4 to 91.4). An interview guide was followed which covered instructors' experiences of the intervention training and using the intervention manual (e.g., “*Were you able to include any of the motivational ideas that were in the manual and training day?”)*, delivering the intervention in a need-supportive way (e.g., *What strategies did you use to motivate the participants?*), their relationship with the participants (e.g., *What was your relationship with the girls like? Did it change?*) and the challenges they faced (e.g., *What didn't work as well during the dance sessions and why?*).

A focus group was conducted with participants from each intervention school (mean duration = 42.4 min, range = 30.4–50.2 min). Girls were sampled to reflect tertiles of intervention attendance within each school to ensure a range of views were elicited (total n = 59; n = 25 high, 16 moderate & 18 low attenders respectively) and focus groups ranged in size from 3 to 8 participants. Of particular relevance to this paper, the focus group topic guide explored participants' views on and relationship with the dance instructor (e.g., *Is there anything you would change about your dance instructor's teaching style?*), and perceptions of autonomy, competence and relatedness (e.g., Autonomy: *Do you think you had some control over what you did?* Competence: *How did you find the dance sessions physically?* Relatedness: *Did your relationships with one another change as the weeks went on?*). Importantly, questions aimed at exploring the theoretical elements focused on broad topics through which participants were given the opportunity to express their views.

#### 2.1.2 Quantitative data collection

##### 2.1.2.1 Observed need-supportive teaching style

Dance instructors' fidelity to the need-supportive intervention was assessed by rating the delivery of four intervention lessons per instructor which were randomly-selected within four intervention blocks (one lesson from each of weeks 5–12, 13–20, 21–29 & 30–36). Instructors wore an audio recording device on their arm with a microphone attached to their clothing. The observation tool developed by Haerens et al. (Haerens et al., 2013) was used. Although the tool was originally designed to rate videos of PE teachers, due to ethical constraints we adapted the method to combine rating of audio recordings of lessons with real-time observation. The instrument contains 21 items measuring *Relatedness support* (5 items), *Structure before the activity* (5 items), *Structure during the activity* (6 items) and *Autonomy support* (4 items). One item (“*encourages pupils to persist*”) was excluded based on low validity within the validation study (Haerens et al., 2013). Fifteen items were rated from the audio recordings as they did not rely on visual observation and five items (e.g., *Is physically nearby the pupils*) were rated by direct observation. The frequency of each teaching practice occurring was rated using a four-point scale (0 = *Never*, 1 = *Sometimes*, 2 = *Often*, 3 = *All the time*) for each 5-min segment of the lesson. Observations and ratings were undertaken by a researcher (JMK) who had discussed the definitions and meaning of the higher order constructs and scale items in depth with (SJS) and undertaken a pilot observation/rating. JMK and SJS listened to the pilot recording separately, then together using the observation tool to guide a discussion of the presence of each teaching behaviour in 5 min segments. To check consistency in interpretation, six audio recordings (excluding the items requiring visual observation) were coded by both (JK) and (SJS). Interrater reliabilities (indicated by intraclass correlations; ICC) (Rabe-Hesketh & Skrondal, 2008) were: *Relatedness support* (ICC = 0.01, poor), *Structure before the activity* (ICC = 0.31, fair), *Structure during the activity* (ICC = 0.57, moderate) and *Autonomy support* (ICC = 0.41, moderate). Internal consistency reliability estimates were as follows: *Relatedness support* (α = 0.58), *Structure before the activity* (α = 0.73), *Structure during the activity* (α = 0.62) and *Autonomy support* (α = 0.60). Only the coding of one researcher (JK) was used in data analysis.

##### 2.1.2.2 Participants' perceptions of dance instructor autonomy support

At the end of the intervention, participants (n = 281, 98.9% of participants randomised to intervention arm) reported their perceptions of the dance instructor's provision of autonomy-support using an adapted version of the Sport Climate Questionnaire (Amorose & Anderson-Butcher, 2007). Six items (e.g., *My Active 7 dance instructor listens to how I would like to do things*) were scored using a 7 point Likert scale (1 = *Strongly disagree* to 7 = *Strongly agree*). Items were averaged to create a perceived autonomy-support score (*α* = 0.94).

### 2.2 Data analysis

#### 2.2.1 Qualitative data analysis

Transcripts of interviews and focus groups were analysed by four researchers (JMK, MJE, TM & SJS) using the Framework Method (Gale, Heath, Cameron, Rashid, & Redwood, 2013) using both inductive (i.e., themes arising from the data) and deductive (i.e., a priori SDT-based themes were interrogated within the data) coding strategies. Deductively, the SDT-related components (i.e., motivation types, psychological needs and instructor teaching style) were defined based on extant literature and these definitions were discussed amongst the group of four analysts. Second, text within the transcripts which related to these definitions (including both positive and negative experiences) were collated. Frameworks created from the initial transcripts formed the basis of analysis for the remaining transcripts and flexible to nuances, new examples and refinements which were discussed at regular group meetings. Third, data relating to SDT were interpreted and refined by SJS and discussed and agreed within the team. A framework for dance instructors and girls was created and a convergence coding matrix which allowed comparison of themes between dance instructors and girls was developed in NVivo (Version 10, QSR International Pty Ltd) (Farmer, Robinson, Elliott, & Eyles, 2006). The application of trustworthiness criteria (Shenton, 2004) included credibility, transferability, dependability and confirmability and are reported elsewhere (Sebire et al.,).

The qualitative data were organised into six themes which combined deductive (e.g., we asked questions about perceptions of relatedness) and inductive (e.g., discussions emerged about challenges of delivering the SDT components) results from the instructors and the girls. The themes were: (1) *Dance instructor training and acceptance of the intervention theory,* (2) *Autonomy support and perceptions of autonomy need satisfaction,* (3) *Dance instructors use of controlling strategies,* (4) *Competence support and perceptions of competence need satisfaction,* (5) *Relatedness support and perceptions of relatedness need satisfaction* and (6) *Challenges of delivering an SDT-based physical activity intervention for children.* Quotes are reported using linked dance instructor and school ID numbers (i.e., Instructor 21 delivered lessons in school 21) and are the same as those used in the other process evaluation papers from this study to facilitate cross-referencing.

#### 2.2.2 Quantitative data analysis

For the observed need-support, scores for each item at each 5-min segment were aggregated to give lesson item mean averages which were then combined to form average scores for relatedness support, structure before the activity, structure during the activity, and autonomy support, for each of the four lessons. Means and SDs for each construct over the four observations were calculated and analysed descriptively. Means and standard deviations (SD) were calculated for girls' perceptions of each of the dance instructor's autonomy supportiveness and were analysed descriptively.

## 3 Results

### 3.1 Quantitative results

**Fig. 1:**
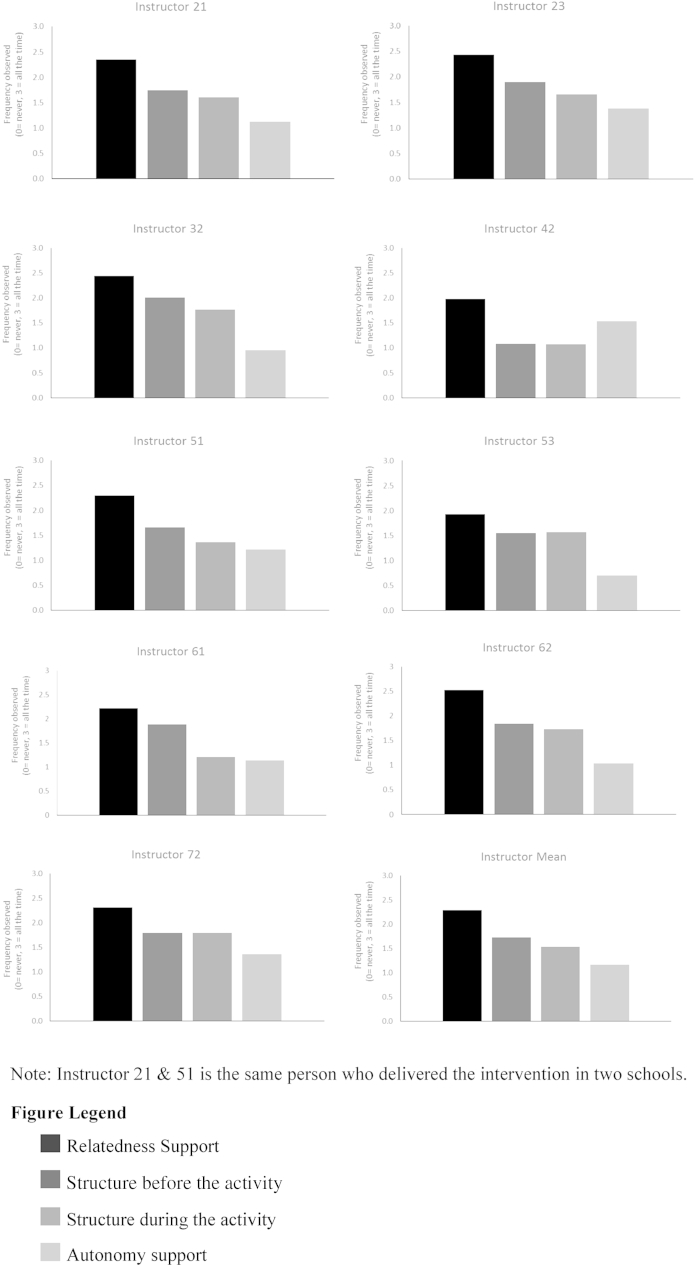
Rated need-support scores for the Bristol Girls' Dance Project dance instructors.

Relatedness support was the most highly scored (between “often” & “all the time”) teaching behaviour amongst all instructors (mean = 2.29, SD = 0.47) (Fig. 1). In general, dance instructors provided moderate (between “sometimes” & “often”) structure before and during the dance activities (structure before, mean = 1.73, SD = 0.54; structure after, mean = 1.53, SD = 0.60). Structure was observed less in the lessons led by instructor 42. Autonomy-support was, for all but one instructor, the lowest scoring teaching practice (mean = 1.16, SD = 0.54) reflecting provision of autonomy support only “sometimes”. Pupil-perceived autonomy support was moderate (mean = 4.68, SD = 1.68) and relatively consistent between instructors (Range: Instructor 51 mean = 4.32 SD = 1.65 to Instructor 21 mean = 5.53, SD = 1.20).

### 3.2 Qualitative results

#### 3.2.1 Dance instructor training and acceptance of the intervention theory

Dance instructors reflected positively on the training and believed that the principles of SDT were appropriate to underpin the dance lessons. Most instructors believed that their existing teaching style was aligned with the SDT approach however one instructor felt that the autonomy-supportive style challenged her existing practice, particularly the language she used:

*A lot of us are quite experienced teaching and you can get into a groove with how you teach and [the introduction of SDT] really made you challenge those sort of key phrases that you say throughout the class.* (Dance instructor 23)

##### 3.2.1.1 Autonomy support and perceptions of autonomy need satisfaction

Dance instructors reported providing girls with choice within dance lessons, including the music, dance styles, choreography and warm-ups which was corroborated by some girls.

*[The dance instructor] asked us what types of things we wanted to do. Some people said contemporary, some people said breakdancing [ … ] so that's what we did, which was good.* (Focus group 53)

Girls choosing the music was an important source of ownership/autonomy within lessons as it made them more engaging and positively influenced activity:

*If it's music they like then they want the music on all the time … they're going to be more active and more involved. It just makes perfect sense to … let them have that choice in the music and it motivates them more.* (Dance instructor 42)

Instructors were encouraged to support participants' autonomy within a clear structure which was developed in the early lessons by involving girls in the development of group rules. They also reported responding to feedback from the girls and attempting to include the views of the group not just a vocal minority (although some children argued to the contrary).

*I would read their [dance] diaries and sometimes they would write things in there, either about the session that would give me clues as to what … you know, 'oh, I loved this game' and you think oh, I didn't realise you loved this game. OK let's do this game more*. (Dance instructor 42)

Generally girls enjoyed the level of autonomy they were granted. However, some stated a need for dance instructors to balance autonomy with sufficient instruction and supervision to support their engagement:

*We had to do it by ourselves and I didn't know the counts or anything and I had to like tell them [others in her group] what to do and I didn't like that …*. (Focus group 61)

##### 3.2.1.2 Dance instructors' use of controlling teaching strategies

While the majority of dance instructors reported using autonomy-supportive styles, participants from several schools (one in particular) described controlling teaching strategies. The following example is where one Dance instructor (DI 53) covered another's (DI 21) lesson:

Participant 5: *It was like the army.*

Participant 2: *She forced you to do handstands.*

Participant 6: *And if you were talking or something she would make you do ten press-ups.* (Focus group 21)

Some girls commented that they “*had no say in pretty much anything*” (Focus group 32) and identified that where choices given they were not perceived as genuine:

*She was asking us to choose a dance and then she'd choose a dance herself.* (Focus group 21)

The frequency and length of drink breaks was considered important to participants' autonomy, but was rarely mentioned by instructors, other than as creating an opportunity for disruption which they had to control by being what participants felt was *“strict”*. Girls rationalised some of their dance instructor's controlling behaviour as being driven by a desire to avoid group arguments or encourage dedication:

*She was strict because she wanted you to be dedicated and turn up.* (Focus group 53)

##### 3.2.1.3 Competence support and perceptions of competence need satisfaction

Instructors reported using numerous competence-supportive teaching strategies including affording participants with the required dance skills, using peer role models, differentiation of dance sequences, encouraging self-reflection, giving opportunities for leadership, and providing constructive feedback.

*I think the easiest way to deal with the [different skill] levels [is to] get those girls who are working really well to help other girls that … are struggling.* (Dance instructor 21 & 51)

Several approaches were used to encourage girls to reflect on their competence, including using the dance diaries, reflecting on their own progress and ensuring that this reflected genuine progress:

*[A participant would say] “I can't do it” and I'm like “well, first of all you can do it”, but also … “remember that step that you couldn't do a couple of weeks ago?” and she's like “oh yeah, I can do it well easy now” and I'm like “well, there you go then” … I'd get them to reflect on their own progress and then I didn't have to try and pretend.* (Dance instructor 21)

Instructors' awareness of girls' abilities allowed them to provide targeted competence support:

*There was a bigger girl who came quite a lot and she found - or I found when I was teaching the structure [choreography] she would struggle and give up a lot easier, because I think she felt like she couldn't do it. She loved teaching the warm-ups and I found that she worked harder, she got sweatier, she pushed herself more because she was confident doing the things that she already knew how to do.* (Dance instructor 53)

Girls corroborated the dance instructors' competence support and reported receiving individual and group-level assistance:

*She helped like if you were stuck on something. But she helps more like as a whole group whereas [a different instructor that the group had] would sort of just help you individually.* (Focus group 23)

Girls reported increased confidence and competence to dance which was also observed by the dance instructors:

*After 30 seconds the first time we were like tired and like couldn't do it. Then after a few sessions … well, not a few but like half way through, we could do it for like ten minutes, five minutes.* (Focus group 53)

In contrast, some participants felt a lack of support when learning more complex skills and thought that the instructor was not aware of the varied competence of the group members. Girls suggested that they could not control the speed at which lessons progressed:

*If we didn't know how to like do the move, like it was a bit hard to ask [dance instructor] to show us to do the move again because she was already showing the next bit.* (Focus group 32)

##### 3.2.1.4 Relatedness support and perceptions of relatedness need satisfaction

All dance instructors referred to using strategies to build trusting relationships with and between girls, including asking them about their lives outside the intervention, responding to comments written in dance diaries, asking after girls when they missed lessons, giving regular high-fives, using a ‘head-to-head sharing time’, and discussing non-attendance:

*I had this one sort of thing where we lie on the floor with all our heads together, and each say one thing about the session that we felt we did really well or it could be one thing that someone else did well …* (Dance instructor 32)

Dance instructors and girls reported feeling a strong trusting relationship which developed over the course of the intervention:

*I had a couple of girls really open up to me and talk to me about sort of personal problems that they were having.* (Dance instructor 23)

*There were a lot of “twelve year old teenage dramas” and people would get upset about 'oh no, my friend doesn't like me, oh!' and then [dance instructor] would be like 'right, we're going to dance this out' [… ] or get them to apologise.* (Focus group 42)

In general, girls considered dance instructors to be enthusiastic, fun and understanding.

*She was really nice because we came in and she was like 'oh, you're the dancers!' We were like 'oh yeah'. And she was really nice. She came in and like introduced herself and everything. And then she … if one … like some of us is like injured or doesn't really want to do dance then she'll let us sit out and then just like come back when we feel like it so she's really nice.* (Focus group 61)

However some girls did not feel a genuine connection with the dance instructor:

*She had to be in charge all the time. If she had kind of like stepped back and been more of a friend than someone like in charge of us then I think we'd have all found it easier.* (Focus group 32)

Where dance instructors' comments or actions were not perceived as genuine, this undermined the participant's connection with them:

*Yeah, [dance instructor] always like really clapped for them. She always clapped for us but like, you know, it was like for them it was, it was like a proper clap.* (Focus group 61)

Girls and dance instructors described the development of existing friendships and the formation of new ones over the course of the intervention: “*We bonded together”* (Focus group 42). At the start of the intervention, many girls were apprehensive and groups were fractured or consisted of existing cliques. However, throughout the project, these cliques dissolved and participants reported making friends and feeling more socially comfortable.

Participant 1: *In the first two sessions I was really shy and I always went to the back and I didn't really say [ … ] much. But now in the Active7 sessions I talk quite a lot [… ] because I got to know quite a lot of people …*

Participant 2: *You feel more comfortable around them.* (Focus group 72)

However, some girls experienced a lack of connection with their peers which seemed rooted in participants' interpersonal comparisons of their dance abilities and divides between the ‘confident’ and ‘shy’ participants.

*The girls who already do dance are really like strong about it and they always go together in a group, they don't share it.* (Focus group 32)

##### 3.2.1.5 Challenges of delivering an SDT-based physical activity intervention for children

Two issues that appeared to challenge the dance instructors' theoretical fidelity in the intervention were the management of disruptive behaviour and the use of end-of-project performances. Some dance instructors found it difficult to be autonomy-supportive when faced with disruptive behaviour:

*There are times, as I said before, when you've got 25 plus of them all going a bit mental [ … ] then you do have to sort of change tactics unfortunately, but generally speaking it [being autonomy-supportive] would be the way I would want the sessions to be.* (Dance instructor 42)

In one case a dance instructor felt conflicted between maintaining high fidelity (using the SDT-based guidelines) and keeping control of the discipline:

*They were running wild and I was trying to be, you know, use the ABC [Autonomy, Belonging, Competence] and it was very hard to try and keep to that. Really, really difficult because they were just testing my limits and going crazy [ … ] I think that's because I was so worried about sticking to ‘this is what we had to do’ to then kind of trying to actually respond to the children themselves.* (Dance instructor 21 & 51)

Some instructors reflected that more role-play based learning in the training would help prepare them for dealing with this more effectively:

*I would say it would be good to … set up some situations where that skill [referring to need supportive teaching] could be practised because I felt frustrated with myself sometimes that I didn't know [ … ] what to say and I didn't know how to do it.* (Dance instructor 23)

A further challenge to theoretical fidelity concerned the use of dance performances as a motivational tool and whether or not the girls wanted to perform in front of others. Girls were given choice as to whether they performed a dance in front of an audience or not and groups often chose to perform. Dance instructors considered performances to be an important motivational element of a dance programme: *You've almost got to have like an end plan that they're all working towards* (Dance instructor 62). Dance instructors also considered performances to be desired by and a positive experience for most girls, although several reflected on performance-related anxiety:

*Even if they're slightly petrified of it or whatever, you know … it's kind of a good fear [ … ] They did all love doing this as well, even though all moaned a bit but they loved it.***(**Dance instructor 62)

Some girls liked the motivation and focus a performance provided:

*At least it's [a performance] for something [ … ] Because if you know you're not doing it in front of lots of people you kind of lack a bit, but if you know you're doing it for, in front of people then you know that you've got to try and do your best and try and get the steps right.* (Focus group 23)

Whereas for others performing was a source of anxiety:

*When we were doing our performance … [dance instructor] wasn't there like … to support us in a way, she didn't come to it, so like we didn't really know the music and we had to do that ourselves … some people [were] really nervous and [saying] they weren't going to do it.* (Focus group 61)

The performance element also was a source of pressure for the dance instructors and one identified that it negatively affected her teaching:

*One session I had to get a bit strict and ended up getting a bit sort of arsy [… ] I think it was the pressure of the performance.* (Dance instructor 23)

## 4 Discussion

In this paper we report a theory-based evaluation of the BGDP intervention using a mixed methods approach. The results can be used to better understand theoretical fidelity and shed light on the results of the trial.

The majority of instructors believed that the SDT principles of the training aligned with their teaching styles and methods. The training was well received and served as a reminder to instructors to focus on the “how” (i.e., communication practices) in addition to the “what” (i.e., dance content) of their teaching. A previous study among PE teachers (Aelterman et al., 2013) identified similar acceptance of SDT-based intervention training and research has shown that classroom teachers' beliefs that implementing an SDT-based teaching style is likely to be effective, easy and normal/usual are associated with their motivating style (Reeve et al., 2013). We believe that our findings indicate that the dance instructors involved in BGDP did buy-in to the SDT teaching style, and believed that it would be effective however the reality and ease of implementing it in practice with the participant group led to variable fidelity as discussed below.

Dance instructors qualitatively reported providing choice (of music, dance styles, warm-up activities, and choreography) within sessions that reflected examples given in the training manual (this therefore also indicates fidelity) and this was perceived by instructors and girls to make lessons more engaging and enjoyable. However, this provision largely reflected *option choice*, and did not appear to provide *action choice*, such as having control over the pace of task progression which is a central element of autonomy-support (Reeve, Nix, & Hamm, 2003). Previous research suggests that providing action choice promotes self-determination and intrinsic motivation more than option choice (Reeve et al., 2003). Instructors that mainly provided option choices may have believed that this was sufficient autonomy-support and neglected action choice. The quantitative need-support ratings corroborate this finding and suggest low provision of autonomy-support across all instructors and substantial room for improvement. This finding is consistent with Haerens et al. (Haerens et al., 2013) who reported that autonomy-support was the least common need supportive practice amongst PE teachers relative to other practices. Further, the qualitative results highlighted the importance of combining autonomy-support with structure as some participants felt uncomfortable when they were left to practice on their own with insufficient instructions. Previous work has shown that teaching styles which combine autonomy-support and structure are associated with improved learning, behavioural and motivational outcomes amongst adolescents (Vansteenkiste et al., 2012) and highlight the importance of ensuring that interventions are able to help teachers balance these two teaching dimensions.

In addition to instances of low autonomy-supportiveness, the qualitative findings identified that some dance instructors used somewhat controlling motivational practices. Instructors may have used controlling strategies for a number of reasons; first, the intervention training may not have successfully changed their teaching styles to be more need-supportive and they adopted their usual practices which included controlling techniques. However, the instructors reported believing that the philosophy of SDT chimed with their usual teaching practices which suggest that this may not have been the case. The SDT component of the dance instructor training was comparable in duration to previous training for PE teachers (Aelterman et al., 2013), but shorter than others (Aelterman, Vansteenkiste, Van den Berghe, De Meyer, & Haerens, 2014). Instructors reported wanting more time to practice implementing the different motivating techniques, suggesting that the training may have been conceptually clear but not sufficiently practical. Previous studies of SDT-based training have incorporated videos of real teaching scenarios (Aelterman et al., 2013, Aelterman et al., 2014) which can be used to identify and reflect on real teaching practices and dedicated more time to practicing motivating strategies. Second, some dance instructors may have misinterpreted the theory, confusing autonomy-support with a lack of rules or structure. The need-support ratings suggested that structure was used with moderate frequency which provides some evidence for this hypothesis and may have led to more disruptive behaviour and reversion to the use of controlling strategies. Third, some dance instructors may have adopted more controlling practices in response to the challenges associated with teaching large classes of beginners in a school environment. For example, one dance instructor *drifted* (Bumbarger & Perkins, 2008) from the need-support foundation by using press ups as punishment for talking. Others used working towards a dance performance as a motivational lever which while seen positively by some, was also a source of pressure for others and their dance instructors.

Moving beyond dance, to broader physical activity interventions which rely on trainers leading groups of young people, our findings suggest that future work is needed to identify whether and how physical activity intervention deliverers use controlling strategies. Further, and based on the nature of trainer's existing practices, it is important to ascertain whether they can be equipped with techniques to use in response to challenging behaviour without resorting to controlling techniques. The findings highlight a need for those developing theory-based interventions to identify innovative ways to communicate theoretical nuances (e.g., action *vs*. option choice) that can be understood and implemented by practitioners. As theory is sometimes viewed as lacking real world validity (Davidoff et al., 2015, Rothman, 2004), there is a risk that efforts to ensure practitioners adopt theoretical principles result in theoretical dilution and more room for drift (Bumbarger & Perkins, 2008) from the intended theoretical targets.

Embracing technology within theory-based complex physical activity interventions may be an effective way to overcome some of the issues which in our study were commonly derived from having limited time within training to adequately cover detailed and subtle theoretical nuances alongside other content or intervention deliverers facing challenges within lessons. For example, previous work has supported training with self-study websites which teachers are asked to engage with (Reeve, Jang, Carrell, Jeon, & Barch, 2004) which include videos of real teaching scenarios. A possible next step is to develop smartphone apps to support in-person training, extend the possible training time and provide a hub of resources to support intervention fidelity such as multimedia content, tasks which reinforce learning, tips when dealing with challenges & networking with other instructors.

Dance instructors reported confidently providing competence support to girls and used a number of techniques which may have utility in future PA interventions. These included a number of examples of providing structure during the dancing activities such as peer–peer teaching, encouraging self-reflection, providing genuine encouragement and provision of structure through clear instructions before the dance activities. The dance instructors' experience in teaching may explain their confidence in using these strategies and supports the importance of identifying well trained intervention deliverers who can bring beneficial *innovation* to PA interventions (Bumbarger & Perkins, 2008). Girls also had positive experiences of competence support (and some reflected on their own increased levels of perceived competence). However, in the BGDP trial, perceived competence towards dance and physical activity decreased pre-post intervention in the intervention group (Jago et al., 2015). It is possible that both results are correct; that while some participants did feel more competent after the intervention, girls on average did not. Alternatively, the results could point towards changes in the girls' cognitive representations of dance before and after the intervention. Pre-intervention, the majority of girls had no prior experience of formal dance lessons. Dance competence levels were rated as relatively high; which could reflect informal dance experiences (e.g. dancing with friends or alone). However, after experiencing dance in a more structured taught environment, trying more complex choreography and comparing their ability with that of their peers, it is not unreasonable to assume that their cognitive representations of dance and their frame of reference for their perception of competence could have changed post-intervention. This could mask some of the qualitative perceptions of increased competence.

Relatedness support was the most commonly observed need-supportive technique used by the dance instructors which in most cases was verified by the qualitative findings. This is consistent with previous research among PE teachers (Haerens et al., 2013), but the dance instructors in our study provided more relatedness support (2.29/3.00) than previously studied PE teachers (≈1.30/3.00) (Haerens et al., 2013). This may reflect the interpersonal style the dance instructors have developed through their teaching of dance to groups of girls in out-of-school settings and be an indicator that they found this particular dimension of need-supportive instruction easy which has been shown to be associated withteacher's motivating style (Reeve et al., 2013). Previous research suggests that relatedness towards PE teachers is associated with girls' engagement in PE (Shen, McCaughtry, Martin, Fahlman, & Garn, 2012). The qualitative findings identified the development of some strong and trusting girl-instructor relationships. The instructors' use of a number of relatedness-supportive techniques which represent effective intervention *innovation* (Bumbarger & Perkins, 2008) could be adopted in other interventions (e.g., dedicating time at the end of lessons for the instructor and girls to lie on the floor with their heads together and reflect on the lesson, giving regular high-fives, using the dance diary to guide empathically changing lessons in line with what girls enjoy/don't enjoy). However, it was clear from the findings that for a minority of girls, a sense of relatedness was not formed with the instructor which was commonly caused by perceptions that the instructor-participant bond was not genuine. It would be useful in future interventions to identify the use of effective techniques during implementation and share them amongst the network of practitioners who are finding relatedness support difficult. Whilst extra support for instructors was included in BGDP mid-intervention, a more effective dissemination of teaching techniques or more frequent provision of materials/support (e.g., via an intervention resource such as an app as referred to above) could hold promise in challenging low-fidelity during theory-based PA interventions.

### 4.1 Strengths & limitations

The theoretical underpinning of BGDP is a strength that has helped to evaluate where the intervention was consistent with, or drifted from, the intended behaviour change strategies. In addition, the combination of quantitative and in-depth qualitative data collected from both dance instructors and participants has facilitated the development of a detailed picture, strengthened by triangulation between participants and across methods.

An inherent limitation in theoretical process evaluations is that the intervention deliverers are aware of the theoretical foundations with which they are asked to underpin their delivery. As such, there is the potential for their interview responses and observed lessons to be biased towards good fidelity. However the focus group results largely added credibility to the dance instructor results and we are confident that we heard a diverse range of perspectives including negative experiences which we have reported. Furthermore it is unlikely that the instructors would have been able to change their teaching style significantly during the four observations, particularly given that the instructors were informed on the day of the lesson that they would be observed. A related limitation is that the inter-rater reliability of the rated dance instructor teaching styles was low for relatedness support and low/moderate for the other dimensions but generally lower than previous work with PE teachers (Haerens et al., 2013). A potential reason for this is that we rated audio rather than video recordings of the dance lessons (as the original measure used) and thus underestimated the information given by physical indicators alongside the audio to make the ratings. However, we only used the ratings of one observer whose ratings were consistent as indicated by good internal consistency estimates. An additional limitation is that we did not measure the dance instructors' perceptions of using an autonomy-supportive teaching style pre- and post-training. This would have afforded us a short-term check of training effectiveness and could be incorporated into future intervention designs. Further, we used a general measure of girls' perception of instructor autonomy-support, which prevented us from examining individual psychological need support and comparing this to our more nuanced observation and qualitative data. Finally, research including applications in the sport and PE domains (Bartholomew et al., 2011, Haerens et al., 2015) has separated the concept of need thwarting (e.g., a child perceiving that their teacher/coach is trying to control or manipulate them) from the experience of low need satisfaction (e.g., that a child does not feel that they have much input in lessons/training sessions). In PE, pupils' perceptions of their teacher's controlling teaching was associated with their need frustration whereas perceptions of teacher autonomy support were associated with need satisfaction. In sport coaching, after controlling for need satisfaction, adolescent athletes' perceptions of psychological need thwarting have been positively associated with exhaustion and negatively associated with vitality (Bartholomew et al., 2011). In the present study, we did not quantitatively measure need thwarting nor the controlling practices of dance instructors. Future process evaluations of SDT-based PA interventions which involve teachers or coaches would benefit from considering need thwarting alongside need satisfaction.

## 5 Conclusion

It is recommended that complex health behaviour change interventions are based on sound theory and that the theoretical elements are subjected to in-depth process evaluation (Moore et al., 2014). The findings of this theory-based process evaluation indicated that theoretical fidelity within BGDP was variable. We identified a number of instances of high theoretical fidelity and intervention innovation which informs pragmatic techniques that intervention deliverers working with groups of children could use. Illuminating the lack of intervention effectiveness, we also found that there was much room for improvement as we identified examples of low fidelity, some drift from the intended motivational techniques and potential failures to convert theoretical nuances into practice. More broadly, this work has highlighted the value of combining quantitative and qualitative approaches in theory-based process evaluations of physical activity interventions.

## Figures and Tables

**Table 1:** Mapping of self-determination theory-based intervention targets to dance instructor intervention manual and dance lesson content.

Theoretical target	Instructor intervention manual/“*motivation toolkit”*	Dance lesson design and content
Autonomy Support	• Definition & description of autonomy need • Definition & description of autonomy-supportive vs. controlling style • Example autonomy supportive teaching strategies, vignettes • Separation from a laissez-faire style • Using autonomy-supportive vs. controlling language (examples) • Using rewards wisely (unexpected verbal praise) • Instructor learning tasks including case studies & self-reflections.	• Provision of choice on music & dance styles • Developing own dances & sections of dances • Using variety of role models (e.g., leading warm up) • Provide girls the option of whether to build up to a dance performance or not & the nature of any performance
Competence Support	• Definition & description of competence need • Example competence supportive teaching strategies, vignettes • Supporting competence verbally & through the setting/nature of tasks • Instructor learning tasks including case studies & self-reflections.	• Begin with a familiar dance (i.e., the dance from the “taster session”) • Differentiation of activities to varied skill levels • Progression from simple to more complex skills & routines • Dance diaries completed at end of lessons to encourage self-reflection • Allow girls to dictate rate of progression/when to move on
Relatedness Support	• Definition & description of relatedness need • Example relatedness supportive teaching strategies, vignettes • Definition & description of involvement including example strategies • Instructor learning tasks including case studies & self-reflections.	• Dance Instructor asking about girls' lives outside of Active 7 (e.g., weekends, holidays) • Focus on teamwork, group dances & building group ethos
Structure	• Definition & description of structure • Separation from laissez-faire style • Discipline & staying safe (using rationales) • Developing behavioural guidelines/instructor & pupil expectations • Dealing with poor behaviour (stepped approach)	• Involve girl's in setting rules within first two weeks • Remind girls of rules & provide rationales • Implement stepped procedure to deal with poor behaviour
Promoting autonomous motivation	• Definition & description of motivation types & the “Active 7 Zone” (intrinsic & identified) • *“What is wrong with controlled motivation” –* definitions and description.	• Focus lessons on having fun & maintaining energetic teaching. • Avoid/minimise use of external controls (e.g., punishments) or rewards or internalised pressure (e.g., guilt trips)
